# Obstetrician-Gynecologists and Perinatal Infections: A Review of Studies of the Collaborative Ambulatory Research Network (2005–2009)

**DOI:** 10.1155/2010/583950

**Published:** 2010-11-11

**Authors:** Meaghan A. Leddy, Bernard Gonik, Jay Schulkin

**Affiliations:** ^1^Research Department, American College of Obstetricians and Gynecologists, 409 12th Street, SW, Washington, DC 20024, USA; ^2^Department of Psychology, American University, 4400 Massachusetts Avenue, NW, Washington, DC 20016, USA; ^3^Department of Obstetrics and Gynecology, Wayne State University School of Medicine, 6071 W. Outer Drive, Detroit, MI 48235, USA

## Abstract

*Background*. Maternal infection is associated with adverse pregnancy outcomes, and ob-gyns are in a unique position to help prevent and treat infections. *Methods*. This paper summarizes studies completed by the Research Department of the American College of Obstetricians and Gynecologists regarding perinatal infections that were published between 2005 and 2009. *Results*. Obstetrician-gynecologists are routinely screening for hepatitis B and HIV, and many counsel prenatal patients regarding hepatitis B and toxoplasmosis. However, other infections are not regularly discussed, and many cited time constraints as a barrier to counseling. A majority discusses the transmission of giardiasis and toxoplasmosis, but few knew the source of cryptosporidiosis or cyclosporiasis. *Conclusions*. Many of the responding ob-gyns were unaware of or not adhering to infection management guidelines. Obstetrician-gynecologists are knowledgeable regarding perinatal infections; however, guidelines must be better disseminated perhaps via a single infection management summary. This paper identified knowledge gaps and areas in which practice can be improved and importantly highlights the need for a comprehensive set of management guidelines for a host of infections, so that physicians can have an easy resource when encountering perinatal infections.

## 1. Introduction

Pregnant women and their fetuses are at increased risk of complications of viral, bacterial, and parasitic infections. Maternal infection is associated with birth defects as well as adverse pregnancy outcomes, such as intrauterine growth restriction and preterm birth [1] and developmental disabilities [2–4]. For example, cytomegalovirus (CMV) infection can lead to hearing and/or vision loss, as well as cognitive impairment in offspring [5], and human immunodeficiency virus (HIV) can result in infection and illness in the newborn [6]. 

For most infections, effective preventive strategies are available; however, many pregnant women are not aware of the threat of infection nor are they practicing preventive strategies [7–9]. As such, obstetrician-gynecologists should be knowledgeable about these risks and their prevention, and should provide patients with preconception and prenatal counseling, as well as offering appropriate testing, vaccination and treatment.

The American College of Obstetricians and Gynecologists (ACOG) has investigated its physicians' knowledge of perinatal infections, including ACOG and Centers for Disease Control and Prevention (CDC) guidelines, and how they are counseling and managing their pregnant patients. This paper summarizes studies of the Collaborative Ambulatory Research Network regarding bacterial, viral, and parasitic infections published between 2005 and 2009. 

## 2. Methods

### 2.1. Description of the Collaborative Ambulatory Research Network

The Collaborative Ambulatory Research Network (CARN) was created in 1990 to assist in evaluating obstetrician-gynecologists' practices, knowledge, and attitudes regarding a variety of clinical concerns, as well as provide Fellows and Junior Fellows timely information on contemporary practice patterns and elucidate areas in which increased education is needed. This project's aim was to change the way that research was being performed; a majority of information regarding medical practice was based on hospital, as opposed to ambulatory, practice. 

The members of CARN are ACOG Fellows and Junior Fellows who have volunteered to participate in several research studies per year. As of March 2009, there were a total of 1,305 CARN members, 50.4% male and 49.6% female. Male members have a mean year of birth of 1954.2 ± 9.8 and a median year of 1953. Female members have a mean year of birth of 1962.5 ± 8.7 and a median year of 1964. In light of this difference, each of the studies reported controlled for age and gender in analysis and compared these groups to identify and report any differences in knowledge, attitudes, or practice. The mean year of birth of the entire CARN sample is 1958.3 ± 10.1 and the median year is 1959. CARN members are representative of each of the 11 ACOG districts, 10 of which are geographic regions, and one which is made up of the U.S. military. 

For all studies, a computer-generated random sample of nonnetwork Fellows and Junior Fellows is included as a reference group, and both CARN and non-CARN members are mailed surveys simultaneously. Because CARN members typically have a higher response rate, a greater number of non-CARN members are sampled, in order to maximize the probability that the non-CARN group is representative of all practicing ACOG Fellows. Any significant differences between CARN and non-CARN respondents are reported.

### 2.2. General Description of the Survey Questionnaires

Each survey study was approved by an Institutional Review Board and accompanied by a cover letter detailing the purpose of the research and indicating that the return of a completed survey would serve as a consent to participate in the study. 

All ACOG survey studies are designed with the purpose of generating descriptive data regarding obstetrician-gynecologists' contemporary practice patterns, clinical experiences, topical knowledge, beliefs and attitudes, medical school/residency training, access to professional and patient resources, and self-reported educational needs. Demographic questions are included in all surveys, asking respondents' age, year of graduation from medical school, gender, type of practice, racial distribution of patients, and location of practice. Questionnaires have included many types of questions: forced-choice (respondents must select a single answer from options offered), multiple answer (respondents may choose all answers that apply), relative (respondents rank or rate a set of statements), quantitative (respondents must volunteer a number, e.g., percent of patients or number of cases within a specified time period), or open-ended (e.g., describing an aspect of their typical course of treatment). Sometimes scales (e.g., to examine respondents' knowledge or attitudes) are included in the survey. In those cases, a Likert-type scale is used for a set of questions. The answers to those questions can be combined to create a score to investigate more global questions. 

For the current review, all ACOG studies of obstetrician-gynecologists' knowledge, attitudes and/or practice regarding perinatal infections that were conducted since 2005 were included. Each study had a response rate of over 50%, with respondents from each ACOG district, and thus, findings are considered to be representative of nationwide practice.

## 3. Results

Obstetrician-gynecologists can counsel patients on how to prevent infection and can also treat and monitor patients who have an infection. One study [10] investigated obstetrician-gynecologists' practices regarding infections during pregnancy in order to determine the range of infections that are assessed during prenatal care. Ross and colleagues [10] found that 88% of ob-gyns reported testing all pregnant patients for hepatitis B virus. Only 1% reported testing all patients for CMV infection, though 60% tested upon patient request, 60% did so after patient reports of significant exposure, and 44% tested for CMV infection if a fetal anomaly was identified; a similar practice pattern was shown for parvovirus B19 infection [10]. Routine testing for influenza is not recommended, and as such, few (4%) physicians routinely test all patients for influenza. Most commonly, physicians (40%) test for influenza virus after patient report of exposure [10]. Of note, these data were collected prior to the recent novel H1N1 concerns in the United States. Bordetella pertussis is also most commonly tested for after patients report exposure (47%). There are no professional guidelines for lymphocytic choriomeningitis virus (LCMV) screening; however, 30% reported testing after report of exposure, 14.8% upon patient request, and 14.4% in cases of fetal anomaly [10]. 

A majority (67%) agreed that informing pregnant patients about infection risk during pregnancy is a priority in their practice [10]. Though many counsel patients on preventing toxoplasmosis (87.7%), hepatitis B virus (78.8%), and varicella-zoster virus (60%), less than half reported counseling patients on preventing CMV, LCMV, and *Bordetella pertussis* infections. The majority reported counseling typically occurred at initial prenatal exams [10]. 

Physicians are generally knowledgeable of the infections that can result in adverse outcomes for women and/or fetuses and are informed about the behavioral changes that can reduce the risk of infection and discussed them with patients, though some were addressed more commonly than others (see Table 1) [10]. However, despite the fact that obstetrician-gynecologists are knowledgeable of preventive measures, the frequency with which these changes were recommended was lower (see Table 1), with time constraints being a common barrier to counseling [10]. Nearly all (96%) agreed that prepared materials on infections would make counseling easier [10]. 

The study by Ross and colleagues [10] demonstrated that physicians cover a wide breadth of topics related to infections during pregnancy, are knowledgeable of the consequences of diverse infections, as well as means by which they can be prevented. However, the study also revealed that due to time constraints, counseling patients regarding the prevention of infection is often omitted from patient care.

### 3.1. Cytomegalovirus

Cytomegalovirus infection occurs in approximately 1 in 150 live births [11] and can lead to hearing loss, vision loss, and cognitive impairment in about 1 in 750 children [5]. Again, preventive strategies exist to minimize the transmission of this infection. 

Like the study by Ross and colleagues [10], a study specific to CMV [2] revealed that though many physicians are cognizant of behavior changes that reduce infection, fewer are providing this information to patients. For example, 90% of ob-gyns knew hand washing reduces CMV infection risk, though fewer (60%) routinely recommended hand washing to pregnant patients [2]. Similarly, about half identified not sharing utensils (57%) and avoiding children's saliva (55%) as reducing risk, though fewer recommend these behaviors (31% and 30%, resp.). 

Forty-four percent counsel patients about CMV prevention, despite ACOG recommendations that pregnant women be counseled about prevention methods [12]. However, in accordance with ACOG's statement that routine serologic screening of all pregnant women for CMV is *not *recommended, 99% reported they do not perform CMV testing on all patients. Most provide testing after report of an exposure (60.3%), when a fetal anomaly is identified (43.6%), or in response to a patient request (31.8%). Just over one-quarter (27%) reported having diagnosed it in a patient since 2003.

### 3.2. Group A Streptococcus

Group A Streptococcus (GAS) is an uncommon, but serious cause of postpartum and postsurgical infections. Despite the fact that GAS infections are preventable [13], international estimates of GAS infections have ranged from .33 to 3.16 per 1000 births in Jerusalem [14] and were estimated as occurring 1 of every 11,000 live births in the United Kingdom [15].

Van Beneden and colleagues [16] investigated postpartum and postsurgical infections of Group A Streptococcus (GAS). Obstetrician-gynecologists reported both postpartum (3%) and postsurgical (7%) infections. Fewer than one in five (14%) of ob-gyns reported routinely obtaining diagnostic specimens for postpartum infections. This may be due to the fact that physicians are aware that infections are polymicrobial; over half responded that the most common bacterial etiology of intrapartum (57%), postpartum (62%), and postsurgical infections (52%) are polymicrobial in nature. Additionally, many may avoid this diagnostic approach due to the technical issue of obtaining the specimen through a bacterially contaminated portal. Of those who collected specimens, the microbial etiology was determined in only 28% of the samples. In postsurgical cases, microbiologic diagnoses were confirmed in 20%. In 13% of postpartum and 15% of postsurgical infections for which diagnoses were confirmed, GAS was found.

 The majority (73%) of respondents reported that they had not diagnosed any GAS infections. There are no ACOG guidelines regarding GAS, and only 5.6% of respondents stated that they were familiar with the 2002 CDC guidelines for management of GAS infections, and more than 70% were unaware of their existence. However, most (74%) respondents stated that they do follow guidelines regarding diagnosis, management, or treatment of postpartum and postsurgical infections, primarily those put forth by ACOG (59%) and those that are part of hospital infection control protocols (32%) [16].

One important theme of the study by Van Beneden and colleagues [16] was the finding that those who were older and had more years in practice had different practices than those who were younger or with fewer years since residency. Those who were older were more likely to make an effort to determine the etiology of infections. This may be the result of changing medical education and knowledge, or in the epidemiology of these infections.

### 3.3. Human Immunodeficiency Virus

Human immunodeficiency virus (HIV) and the resulting acquired immune deficiency syndrome (AIDS) are pandemic and are associated with severe morbidity and mortality. In the United States, women are reportedly the fastest-growing subgroup with new HIV diagnoses [17]. Obstetrician-gynecologists are in a unique position to provide HIV testing and one concern relevant to obstetrician-gynecologists is the transmission of HIV from mother to fetus. As such, numerous professional organizations recommend that routine prenatal care include HIV testing [17–20].

 Gray and colleagues [21] found that 97% of ob-gyns adhere to ACOG guidelines [22] and recommend HIV testing to all pregnant patients; ACOG recommends that this be done as early in the pregnancy as possible and with the opt-out method of screening where legally possible [22]. Most (83.3%) of those who see pregnant patients reported feeling at least moderately knowledgeable regarding HIV in pregnancy, and 92% reported maintaining contact with an infectious disease specialist for consultation [21]. Respondents most commonly (79%) reported that patients refused HIV testing due to viewing themselves as low risk [21]. When a patient declines testing, 28.7% do not reoffer testing [21]. ACOG recommends that HIV testing should be reoffered or repeated in the third trimester for women in areas with high HIV prevalence, women known to be at high risk for acquiring HIV infection, and women who declined testing earlier in pregnancy [22]. 

 If a patient arrived at labor and delivery with an unknown or undocumented HIV status, 56.3% would act in accordance with ACOG recommendations and conduct rapid HIV testing, whereas 18.2% would not [22]. One quarter (25.5%) stated rapid HIV testing at labor and delivery is not available [21]. Testing strategies (opt-out versus opt-in approaches) were not consistent with state regulations: 57% use the approach required by their state, and 43% reported using a method not approved by their state; 33% did not know if their state had regulations requiring physicians to recommend HIV testing during pregnancy [21]. 

A more recent study [23] investigated patient perceptions of HIV testing. Two-thirds (65%) of patients reported having been tested for HIV at some point in the past, though 72% did not recall if their current ob-gyn recommended testing [23]. Among pregnant patients, 61% did not recall their current provider having recommended testing, though 82% had been tested at some point, with 71% receiving their test results during the current pregnancy [23]. Young, pregnant, Hispanic and African-American patients were most likely to report that HIV testing had been recommended by their obstetrician-gynecologist. Similar to the perceptions of obstetrician-gynecologists [21], patients indicated they most commonly decline testing because of seeing themselves as low risk [23].

The results of these two studies indicate that physicians must be made more familiar with state regulations regarding HIV testing during pregnancy, and communication between obstetrician-gynecologists and patients must be improved in order to discuss HIV testing.

### 3.4. Listeriosis

If pregnant women have Listeriosis (*Listeria monocytogenes*), they can develop severe bacteremia that can spread to the meninges, lungs, liver, lymphatic system, and placenta. Pregnant women are 20 times more likely than healthy adults to contract this infection [24]. Perinatal infection can lead to granulomatosis infantisepticum, a disseminated infection that usually results in intrauterine death. Neonatal listeriosis may present as early onset neonatal sepsis in the first week of life, or as late-onset meningitis after the first week, similar to the presentation of invasive Group B streptococcal infection [25, 26]. Given these severe risks, obstetricians are in a unique position to educate their patients regarding this illness and its prevention, though it should be mentioned there are currently no guidelines regarding this illness. 

In 2002, ACOG and the CDC collaborated to assess a nationwide sample of pregnant women regarding their knowledge of Listeriosis, and the Minnesota Department of Health conducted a similar study limited to pregnant women within that state; the data were presented and compared by Ogunmodede and colleagues [9]. A general lack of knowledge about Listeriosis among pregnant women was demonstrated. For example, less than 30% in both samples knew that Listeriosis could be prevented by avoiding deli meats, soft cheese (e.g., feta, brie), and unpasteurized dairy products [9]. Less than 20% actually avoided delicatessen and ready-to-eat foods while pregnant [9]. In fact, less than 20% had ever read, heard, or seen any information about Listeriosis [9]. Listeriosis has significant maternal and fetal ramifications, and efforts should be made to educate patients about Listeriosis and its prevention.

### 3.5. Parasitic Disease

In addition to viral and bacterial infections, there are diverse food- and waterborne parasitic diseases that can adversely affect pregnant patients. One comprehensive study [27] focused on obstetrician-gynecologists' practices and knowledge of eight food- and waterborne parasitic diseases: toxoplasmosis, cryptosporidiosis, giardiasis, amebiasis, cyclosporiasis, trichinellosis, ascariasis, and taeniasis. Respondents were most knowledgeable about toxoplasmosis; for example, 90.2% knew this illness causes severe ocular and neurologic disease in the fetus, and 77% knew a stool sample does not help diagnose toxoplasmosis [27]. However, only 28.2% correctly indicated that cryptosporidiosis is most likely to be contracted from contaminated water, and only 19.3% identified that cyclosporiasis outbreaks have been associated with raspberries [27].

Knowledge of treatment for parasitic illness was lacking; only 10.1% correctly identified Paromomycin as the safest medication to treat giardiasis in the first trimester, and only 28% knew that special laboratory testing is needed to differentiate the pathogenic *Entamoeba histolytica *(the cause of amebiasis) from the nonpathogenic *E. dispar* [27]. While 67.9% recognized that invasive amebiasis in *non*pregnant patients should be treated with metronidazole and then paromomycin, only 39.6% knew that trimethoprim-sulfamethoxazole, metronidazole, and amoxicillin were not the recommended treatment for cryptosporidiosis in pregnant women [27].

### 3.6. Giardiasis

Giardiasis is a parasitic disease caused by the protozoan *Giardia intestinalis* (*Giardia lamblia*) [28], and its prevalence in industrialized countries ranges from 2% to 5% and upwards of 20% to 30% in developing countries [29]. This parasitic disease can be especially problematic for pregnant women; malabsorption and diarrhea can be harmful to the fetus [30], but, at the same time, some medications may have side effects that affect fetal development [28]. Therefore, ob-gyns can play a role in identifying and appropriately treating giardiasis in women. A study by Krueger et al. [31] found that a majority of ob-gyns provided correct information regarding the transmission of the diseases, prevention methods, and disease outcomes. 

Response patterns indicate that physicians can benefit from further education regarding the treatment of giardiasis. Under half (45.7%) correctly indicated that Tinidazole, Metronidazole, and Nitazoxanide can all be used to treat giardiasis, and 49.6% believed that only Metronidazole was used to treat giardiasis [31]. Additionally, only 16.2% were able to correctly identify that Paromomycin is the safest drug treatment to use during the first trimester [31]. Only a third (33.2%) correctly indicated that it is not usually recommended that asymptomatic carriers be treated [31]. Given the fact that ACOG has not published guidelines regarding Giardiasis and the results of the study by Krueger et al. [31], it is important that information regarding this illness be disseminated to clinicians.

### 3.7. Toxoplasmosis

*Toxoplasma gondii*, a protozoan parasite, can infect humans through undercooked meats, ingestion of contaminated water or soil, and cat or cat litter contact [32–34]. Toxoplasmosis can cause neurological and ocular damage to the fetus if a woman becomes newly infected during pregnancy [35]. Though it is not particularly common, it is important that physicians be cognizant of the prevention, diagnostic, and treatment practices related to toxoplasmosis. An ACOG Practice Bulletin states that pregnant women should be counseled about methods to prevent acquisition of toxoplasmosis during pregnancy [12]. About half (53.4%) of ob-gyns report counseling women on prevention of toxoplasmosis at the initial prenatal visit, and 3.3% reported never doing so [36]. Physicians reported discussing the following means of transmission with patients: handling cat litter (99.6%), inadvertent contact with cat feces (98.0%), eating undercooked meat (77.6%), handling raw meat (67.4%), gardening (65.4%), and washing fruits and vegetables (34.2%) [36]. 

Most (73.2%) ob-gyns were unsure if there are problems with commercial *Toxoplasma* IgM tests, despite reports of high false-positive rates [37], but 68.8% reported they would refer a patient with a positive test to a specialist. Almost all (91.2%) had not heard of the avidity test, a test that can help determine the timing of infection relative to pregnancy [36].

Ob-gyns should counsel patients about the risks of toxoplasmosis and prevention methods. Additionally, they should be aware of the avidity test, as well as the false-positive rates of certain commercial IgM tests in order to interpret them appropriately, and perhaps complete a second test before moving on to more invasive procedures (e.g., amniocentesis).

## 4. Discussion

Common parasitic, viral, and bacterial infections are likely to be encountered in regular obstetric and gynecologic practice. Infections can have an impact on both mother and fetus, and many are preventable either through avoiding common transmission pathways, vaccination, or hand washing. Obstetrician-gynecologists are in a unique position to inform both pregnant women and mothers about the spread of these illnesses, the potential consequences for both mothers and babies, and prevention measures. Basic knowledge of these diseases will improve physicians' ability to identify and treat parasitic infections, which will minimize the risks of harm to women and fetuses. This study is important in that it reviews a half-decade of research on the topic of infections in obstetric and gynecologic practice, highlights improvements to be made, and suggests methods by which guidelines can be better disseminated. 

Results demonstrate that some infections (e.g., hepatitis B) are screened for routinely by obstetrician-gynecologists. In a similar vein, many counsel patients on a variety of infections (e.g., toxoplasmosis, hepatitis B), especially during prenatal exams, but there are some that are not regularly discussed (e.g., CMV, LCMV, and *Bordetella pertussis*). Physicians are also not commonly identifying the etiology of intrapartum, postpartum, and postsurgical infections. While this may be due to the likely polymicrobial nature of infections or that identification of bacteria rarely changes first-line antibiotic treatment, pretreatment cultures facilitate management of patients who fail initial empiric antibiotic treatment [38]. Knowing the cause of an infection allows for antimicrobial treatment to be appropriately tailored; this is especially important when considering that the primary causative infection agents change over generations. For example, the principal etiology of early onset neonatal sepsis has ranged from GAS in the 1930s–1940s, *E. coli *in the 1940s–1970s, to group B streptococci more recently [39]. Further, obtaining pretreatment cultures allows for specific infection control measures to prevent the spread of disease. 

Some improvement can be made in regards to physician knowledge of certain illnesses; although a majority knew and discussed the transmission of giardiasis [31] and toxoplasmosis [36], only a minority knew that cryptosporidiosis is a waterborne illness, and few knew that cyclosporiasis is associated with raspberries [27]. Obstetrician-gynecologists are knowledgeable of the adverse outcomes associated with infections and are aware of preventive measures [2, 10, 27], though, again, some were discussed more commonly than others [2, 10]. Time constraints were commonly cited as hindering one's ability to counsel patients about prevention, and it was indicated that prepared materials would facilitate such discussions.

In order to assist physicians, many guidelines for managing infections have been created; however, there is some evidence that they are not being widely disseminated. For example, almost three-quarters were unaware of the existence of CDC guidelines for GAS infection management [13], and only about half adhere to ACOG guidelines and counsel patients about CMV (44%) [10] and toxoplasmosis (53.4%) prevention [36]. Numerous professional organizations recommend that routine prenatal care should include HIV testing [17–20], and obstetrician-gynecologists are practicing accordingly [21]. However, testing strategies (opt-out versus opt-in approaches) were not wholly consistent with state regulations. In fact, 43% reported using a method not approved by their state, and a third did not know if their state had regulations requiring physicians to recommend HIV testing during pregnancy [21]. Similarly, only half of respondents would act according to ACOG recommendations and conduct rapid HIV testing if a patient arrived at labor and delivery with an unknown or undocumented HIV status [22]; one quarter said this option was not available at labor and delivery [21].

There are some topics for which ACOG has not published guidelines (e.g., GAS, Giardiasis, Listeriosis), which may result in the knowledge gaps demonstrated, particularly for Giardiasis [31]. Guidelines on these topics can provide physicians with information regarding best clinical practice, which may increase discussion of such topics with patients, as well as patients' subsequent knowledge of infection prevention which was shown to be weak (e.g., Listeriosis) [9]. Further, it may be important for organizations like ACOG or the CDC to create a comprehensive summary of prevention and treatment guidelines for physicians, such that they can access a simple reference readily and make informed decisions. 

An additional tool that may minimize the time burden and knowledge gaps and aid physicians in counseling patients regarding infection prevention is the development of automated, computer-based, patient-centered, self counseling. These programs would allow for patients to learn about infectious disease issues on their own time, at their own pace, and become informed about prevention and consequences. 

As with all research, this study has several limitations. Firstly, all surveys were completed retrospectively, subjecting them to possible recall bias. Physicians may be less accurate in their estimates of how frequently they provide various clinical services. Additionally, there is the possibility that a social desirability effect influenced physicians' responses. Participants may have selected responses they believed to be socially acceptable or which presented them in a more favorable light. For example, physicians may over-report the frequency with which they counsel patients regarding certain topics. Efforts were made to minimize this effect by stressing confidentiality and anonymity, as well as excluding identifying information from the measures. A final limitation of this review is that it has been limited only to topics covered by previous research; more common issues (e.g., group B streptococcus, sexually transmitted infections) have not been investigated, and so could not be reviewed here.

## 5. Conclusions

Obstetrician-gynecologists are knowledgeable regarding the prevention, transmission, and management of a variety of perinatal infections, though improvements can be made regarding the information and counseling they provide to patients. In particular, efforts should be made to increase the dissemination of guidelines for infection prevention and management, as well as state regulations regarding HIV testing in order to create uniform practice. Evidence-based guidelines should be created for new areas, and physicians should strive to be aware of recommendations put forth by other organizations; perhaps joint CDC-ACOG guidelines would be of benefit, especially if a single summary of management guidelines was created. Additionally, efforts may be well guided to distribute prepared materials about infectious diseases for patients, as obstetrician-gynecologists reported that such materials would mitigate the impact of time constraints.

## Figures and Tables

**Table 1:** Percentage of CARN respondents who reported knowing that various behaviors can prevent infection, and of those who reported knowledge of the preventive behavior the percent who reported recommending the behavior to reduce the risk of infection during pregnancy.

Infection Prevention Behavior	Knowledgeable that behavior prevents infections (*n*)	Of those knowledgeable of preventive behavior, the percent who recommend behavior to patients (*n*)
Avoiding cleaning cat litter boxes	98.1% (262)	97.7% (257)
Cooking meat until well done	92.0% (262)	82.2% (241)
Getting tested for HIV	91.6% (262)	97.1% (240)
Avoiding contact with people who have chickenpox or pertussis	90.8% (262)	87.0% (230)
Keeping up-to-date on vaccines	89.7% (262)	89.4% (235)
Hand washing after diaper changing	89.7% (262)	64.7% (235)
Avoiding wild or pet rodents	88.5% (262)	63.8% (232)
Not sharing utensils with toddlers	58.8% (262)	40.9% (254)
Not getting children's saliva in eyes or mouth	55.0% (262)	20.8% (144)
